# Fasting-mimicking diet counteracts gut microbial dysbiosis in experimental lynch syndrome

**DOI:** 10.1186/s40170-026-00446-1

**Published:** 2026-06-30

**Authors:** Lorena Garcia-Castillo, Giulio Ferrero, Olga Blaževitš, Giulia Francescato, Azra Thaseen Eliass, Natalia E. Cortez, Marc Beltrà, Sonia Tarallo, Barbara Pardini, Paola Costelli, Alessio Naccarati, Valter D. Longo, Fabio Penna

**Affiliations:** 1 INSERM, MMG, Marseille Medical Genetics, Aix-Marseille University, Marseille, 13385 France; 2 Department Of Clinical and Biological Sciences, University of Torino, Turin, Italy; 3 Italian Institute for Genomic Medicine (IIGM), C/O IRCCS Candiolo, Candiolo, Turin, Italy; 4 IFOM ETS, The AIRC Institute of Molecular Oncology, Milan, Italy; 5 Longevity Institute, Davis School of Gerontology and Department of Biological Sciences, University of Southern California, Los Angeles, CA USA; 6 Institute for Research in Biomedicine (IRB Barcelona), The Barcelona Institute of Science and Technology, Barcelona, Spain; 7 Departament de Bioquímica i Biomedicina Molecular, Facultat de Biologia, Universitat de Barcelona, Barcelona, Spain; 8 Candiolo Cancer Institute, FPO-IRCCS, 10060 Turin, Italy

**Keywords:** Colorectal cancer, Fasting-mimicking diet, Gut microbiota dysbiosis, Microbial pathways, Lynch syndrome

## Abstract

The development of colorectal cancer (CRC) is largely influenced by hereditary factors, with up to one-third of cases linked to genetic predisposition. In parallel, environmental factors such as diet and intestinal microbiota play a significant role. Lynch syndrome (LS), the most common form of hereditary CRC, is due to mutations in DNA mismatch repair genes. Diet interventions such as calorie restriction (CR) can modify the course of the disease, altering nutrient supply and promoting beneficial microbial populations. Fasting-mimicking diets (FMD) are plant-based CR regimens that showed promise in modulating the gut microbiota and suppressing CRC progression in pre-clinical ectopic cancer models. In this study, Villin-Cre/Msh2-floxed (VCM) mice, modelling LS, were subjected to periodic FMD cycles for 10 months. FMD regimen influenced animal weight in a sexually dimorphic manner, stably reducing animal body weight only in males. Moreover, shotgun metagenomic sequencing revealed that FMD mitigated the dysbiotic longitudinal changes associated with cancer onset, preserving beneficial species, such as *Lactobacillus johnsonii*, and reducing adverse species, such as *Escherichia coli*. Metabolic pathway analysis also showed significant differences, with FMD preventing the upregulation of pathways involved in amino acid and nucleotide synthesis, potentially promoting tumour growth. Overall, the findings suggest that periodic FMD may result useful in a multimodal approach for LS management, counteracting gut microbiota alterations.

**Supplementary Information:**

The online version contains supplementary material available at https://doi.org/10.1186/s40170-026-00446-1.

## Introduction

Colorectal cancer (CRC) is the third most diagnosed cancer in the world and represents the second leading cause of cancer deaths [1, 2]. Currently, it is estimated that 10–35% of CRCs are linked to a hereditary predisposition, with a specific genetic cause identified in 3–5% of these cases [3, 4]. The most frequent form of hereditary CRC is known as Lynch syndrome (LS) [5]. LS, also referred to as hereditary nonpolyposis CRC (HNPCC), is characterized by an autosomal dominant inheritance pattern that significantly increases the risk of developing colorectal and endometrial cancers, among others. It is caused by a mutation in one of the DNA mismatch repair (MMR) genes *MLH1, MSH2, MSH6, PMS2,* or a deletion in the 3’ region of the *EPCAM* gene. A defective MMR pathway causes microsatellite instability (MSI), which leads to the early onset of colorectal neoplastic lesions [1, 3, 5]. In addition to hereditary predisposition, environmental factors such as dietary habits, sedentary lifestyle, obesity, alcohol consumption, and intestinal microbiota significantly aid in the development of sporadic CRC [6, 7].

Beyond intrinsic enterocyte genetic alterations driving tumorigenesis, several studies have demonstrated a correlation between gut dysbiosis and the risk of onset and progression of sporadic CRC [8–11]. Indeed, specific bacteria, including *Fusobacterium nucleatum*, *Escherichia coli*, *Bacteroides fragilis*, and *Streptococcus gallolyticus,* have been associated with colon tumorigenesis [12]. Mechanistically, one of the most common pathways in CRC development involves the inflammatory and immune responses triggered by the interaction between CRC-promoting bacteria and the host [12]. Additionally, microbe-derived metabolites in the intestine can act as genotoxic agents by promoting inflammation and DNA damage in the colon, thereby altering genome stability [13]. For instance, secondary bile acids, such as deoxycholic acid (DCA), have been shown to enhance the development of CRC in a cancer-resistant mouse strain after treatment with azoxymethane [14]. In another study, higher levels of DCA were observed in the serum of men with colorectal adenomas compared to healthy subjects [15]. In addition, the presence of colibactin-producing *E. coli pks+ (polyketide synthases)* in the colon has been shown to accelerate mutations and the relative contribution of mutational signatures associated with MMR deficiency [16, 17].

Dietary habits affect the composition and metabolism of the gut microbiota. In this respect, the availability of undigested food influences the abundance, diversity, function, and balance among gut microbial species [18, 19]. Calorie restriction (CR) and fasting may alter the gut environment by modifying the nutrient supply and energy sources, which can directly influence the growth of specific microbial taxa and the production of microbiota-derived metabolites [20]. In mice subjected to nutrient deprivation for 24 hours, the abundance of *Akkermansia, Parabacteroides,* and *Muribaculaceae* increases, while the populations of *Lactobacillus* and *Bifidobacterium* decrease. Interestingly, this modulation is reversed upon refeeding [21]. Given that CR impacts the microbiota and, in parallel, interferes with CRC tumorigenesis, the two events may be causally linked; however, information on this area remains limited. So far, preclinical data have shown that increased *Lactobacillus* [22] and *Bifidobacteria* [23] in the gut during CR have key roles in CRC suppression.

The fasting-mimicking diet (FMD), a plant-based, calorie-restricted regimen, was designed to achieve the benefits of fasting while providing micronutrient nourishment and minimizing the stress associated with fasting [24]. In a murine model of inflammatory bowel disease (IBD), FMD reduced systemic inflammation and increased *Lactobacillaceae* levels approximately 45-fold compared to standard chow [25]. In a recent study, the increase of *Lactobacillus johnsonii* in the intestine of animals subjected to FMD was essential for the anticancer effect of FMD in an ectopic CRC model [26]. In another study, FMD fed CRC mouse models, the diet promoted the increase of *Bifidobacterium pseudolongum,* whose metabolite L-arginine induced an increase of tissue-resident memory CD8+ T-cells [27].

Here, we explore the effects of FMD on the gut microbial population before and after tumour onset in an experimental genetic model of spontaneous intestinal tumorigenesis. Our findings suggest that periodic cycles of FMD, although not affecting tumour appearance, counteract the gut microbiota alterations observed during CRC progression.

## Methods

### Animal model and experimental design

The study was performed in accordance with the ethical standards of the Helsinki Declaration and all the experimental animal procedures were performed in compliance with the Italian Ministry of Health Guidelines, the ARRIVE guidelines, the U.K. Animals (Scientific Procedures) Act, 1986 and associated guidelines, the EU Directive 2010/63/EU for animal experiments. The Bioethical Committee of the University of Torino approved the experimental protocol. The animals were maintained on a regular 12:12-hour dark-light cycle with controlled temperature (20–23°C) and humidity (40–60%).

The Villin-Cre/Msh2loxP/loxP (VCM) mouse model was developed by crossing B6. Cg-Tg (Vil1-cre) 997Gum/J (Villin-Cre) and B6. Cg-Msh2tm2.1Rak/J (Msh2loxP) mice were purchased from The Jackson Laboratory (Bar Harbor, ME, USA). The offspring bears a conditional knockout of the *Msh2* gene in enterocytes, accelerating the spontaneous formation of intestinal adenomas and adenocarcinomas [28]. The presence of each transgene construct was assessed on DNA extracted from the distal tail tip (2 mm) of conscious, non-anesthetized animals [29] using Melt Curve Analysis (RT-PCR) with the following primers: Villin-Cre (forward: 5′-TTCTCCTCTAGGCTCGTCCA-3′ and reverse: 5′- CATGTCCATCAGGTTCTTGC-3′) and Msh2loxP (wild-type: 5′- GATGATGTGTGAAGCCTGCAT-3′, mutant: 5′-CCTCTTGAGGGGAATTGAAGT-3′ and common: 5′-AGGTTAAAAACCAGAGCCTCAACT-3′).

**Fig. 1:**
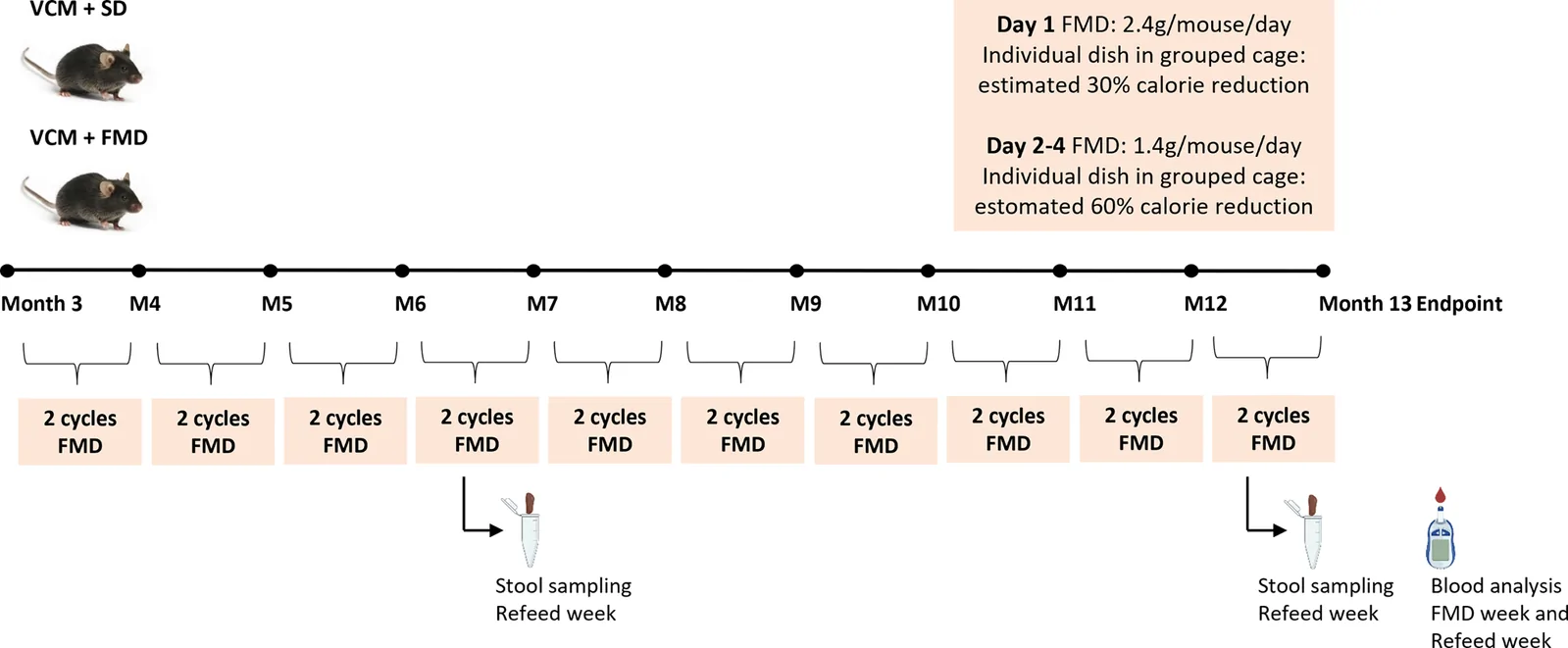
Schematic representation of the experimental design. The cartoon illustrates the study’s experimental plan, which includes the Fasting-mimicking diet (FMD) protocol (administered twice a month until the endpoint), faecal collection, blood ketone and glucose analysis, and the 13-month sacrifice time point. The experimental groups consisted of: 1) male VCM mice fed with standard diet (SD) (*n* = 12); 2) female VCM mice fed with SD (*n* = 12); 3) male VCM mice fed with the FMD (*n* = 14); 4) female VCM mice fed with the FMD (*n* = 12)

After reaching three months of age, male and female VCM mice from different litters were randomized and divided into four groups: 1) VCM fed with standard diet (SD; hereby called VCM+SD) males and 2) VCM+SD females, 3) VCM fed with FMD (from hereby called VCM+FMD) males and 4) VCM+FMD females. The endpoint was set at ~ 13 months of age, a time point when, in general, more than 80% of VCM mice show macroscopic tumours [30]. Hence, animals underwent ~20 cycles of FMD over a ten-month experimental period. Not all the animals were included simultaneously in the experimental set-up; instead, they were included as they reached three months of age from our in-house breeding colony. Body weight and food intake were recorded on a regular basis. Blood glucose and ketones were assessed when the animals reached twelve months of age (Fig. 1). Faecal samples were collected at two time points (always between 3 and 5 pm): T1 (corresponding to 6 months of age) and T2 (at 12 months of age). Mice were examined daily for signs of distress and eventually euthanized when they reached established humane endpoint. The endpoint criteria included pronounced body mass loss (>30% of maximum body weight) and overall condition of the mouse (lack of grooming, kyphosis, inactivity, reduced reactivity, and anorexia).

### FMD diet

In humans, the FMD is designed to provide sufficient nourishment during periods of low caloric intake, consisting of a 5-day regimen, as described by Brandhorst et al., 2015 [31]. In our study, the FMD regimen was adapted for 4 days of diet as the animals achieved a ~ 10–20% body weight loss by the fourth day (Fig. 1). On day 1, the animals housed in grouped cages received individual dishes containing 2.4 g of FMD per mouse to adapt to a period of low caloric intake. On days 2–4, the animals received individual dishes containing 1.4 g FMD per mouse per day. Such feeding scheme was aimed to produce a ~ 30% and ~60% calorie reduction on days 1 and 2–4, respectively, provided that precise monitoring of individual food intake was not performed. The mice showed no signs of food aversion. Before supplying the FMD, the animals were transferred into fresh cages to avoid coprophagy and/or feeding with a residual chow diet. After the end of the FMD cycle, the animals were provided with a standard laboratory chow (SD, Special Diets Services; VRFI) *ad libitum* before starting another FMD cycle.

### Assessment of blood ketones and glucose levels

To assess the proper intake of the FMD regimen, blood glucose and ketone levels were evaluated in a small amount of peripheral blood obtained by removing the distal tail tip (2 mm) from conscious, non-anesthetized animals [29]. Tail tip sampling is a minimally invasive procedure and was carried out by snipping less than 2 mm of tissue from the tail tip with sharp scissors. Blood was obtained by direct flow or by gently massaging the tail and then collecting the blood directly on a glucose or ketone test strip (Nova Pro device). FMD diet administration and blood collection was always performed starting from 4:00 PM within a 1-hour interval, corresponding to Zeitgeber Time 9 (ZT9), thus in the more reasonable time of spontaneous fasting.

### Free fatty acid assay

The concentration of free fatty acids (FFA) in plasma was quantified using a commercial kit (MAK044, Sigma) according to the manufacturer’s instructions. All samples, blanks, and standards were prepared in duplicate.

### DNA extraction and shotgun metagenomic library preparation

Stool DNA extraction was performed with the DNeasy PowerSoil Pro Kit (Qiagen) according to the manufacturer’s instructions. Libraries were prepared starting from 24 ng of faecal DNA using the Illumina DNA Prep (M) Tagmentation kit (Illumina), as described in [32, 33]. The procedure followed the manufacturer’s protocol, except for two in-house modifications as performed by Thomas et al., 2019 [33]: 1) a final clean-up of the pool to be sequenced was performed with 0.6X AMPure XP beads (Beckman-Coulter), and 2) the final library pool was resuspended after the clean-up with 1/3 of the initial pool volume. These adjustments allowed us to retrieve higher-quality reads with a lower adapter content. The sequencing on the NovaSeq 6000 System was performed at the Italian Institute for Genomic Medicine (IIGM) sequencing facility.

### Shotgun metagenomic data analysis

Sequenced metagenomes were pre-processed using the pipeline available at https://github.com/SegataLab/preprocessing for: i) removal of low-quality reads (quality Q < 20), too short fragments (length < 75 bp), and reads with two or more ambiguous nucleotides; ii) host and contaminant DNA removal using Bowtie 2.77 (–sensitive-local) for filtering the phiX174 Illumina spike-in and mice-associated reads (mm10 assembly); iii) creation of paired forward and reverse and unpaired reads output files. On average, 76.66 ± 19.98 million paired-end reads were generated for each sample, of which 67.37 ± 20.53 (87.33±10.36%) passed the quality check and were used for the downstream analyses.

MetaPhlAn 4 (version 4.1, database vJun23, with the “–statq 0.1”) [34] and HUMAnN 3.9 [35] profiling tools were used to define the microbial taxonomic and functional profiles, respectively.

Microbial diversity analysis was performed using the functions implemented in the vegan v2.6.4 R package, while differential abundance analysis was performed with SIAMCAT v2.4 [36]. The analysis was performed on Species-level Genomic Bins (SGBs) by removing the lowly abundant SGBs (using the nearZeroVar function of the caret R package) and those without a defined genus annotation.

### Statistical analyses

R v4.3.1 was used for statistical analyses and graphical representation of the results. Non-parametric tests were performed using the Wilcoxon Rank-Sum method. Multidimensional scaling (MDS) statistical analysis was performed using the Bray-Curtis dissimilarity index, as implemented in the Vegan R package. Paired differential analyses were conducted among time points, while comparisons between SD and FMD animals were adjusted for sex. A test was considered significant if it was associated with a *p*-value of less than 0.05.

## Results

In this longitudinal study, VCM mice were subjected to periodic cycles of FMD for ten months (experimental protocol depicted in Fig. 1). VCM mice develop heterogeneous and non-synchronized tumours; thus, not all mice reached the established 13-month endpoint or developed macroscopic tumours at the endpoint. However, the sample size was not powered to draw conclusive evidence on the effect of FMD on tumour incidence in VCM mice. In the VCM+SD group (12 males and 12 females), 8 males and 6 females showed macroscopic tumours at the endpoint or died prematurely, likely due to tumour presence. In the group subjected to FMD (14 males and 12 females), a total of 12 males and 7 females exhibited the presence of tumours at the endpoint or died prematurely (Supplementary Table 1).

### FMD impact on systemic metabolism of VCM mice

**Fig. 2:**
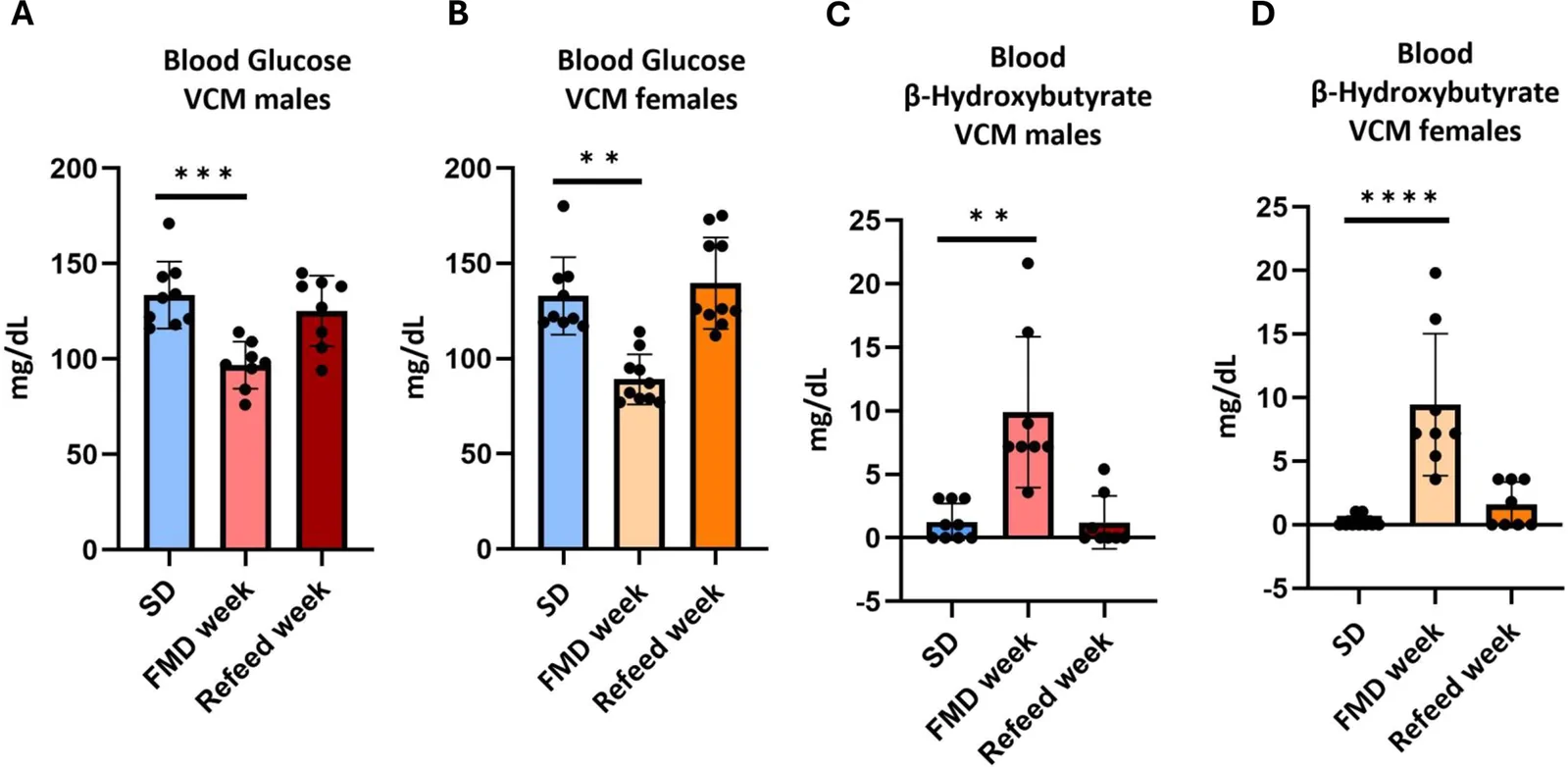
Blood glucose and β-HB levels in VCM mice. A-B) blood glucose levels (means ± SD) expressed in mg/dL on the last day of the FMD week or one week after refeeding in males (**A**) and females (**B**). C-D) blood β-HB levels (means ± SD) represented as mg/dL on the last day of the FMD week or one week after refeeding in males (**C**) or females (**D**). VCM+FMD males (*n* = 9) and females (*n* = 10). Mice that reached the endpoint are represented. Student’s t-test: *p < 0.05; **p < 0.01; ***p < 0.001

FMD stimulated molecular hallmarks of fasting, such as low blood glucose levels and increased ketone bodies in circulation. To shed light on the metabolic shift caused by FMD, blood glucose and β-hydroxybutyrate (β-HB) levels were assessed in mice at twelve months of age (Fig. 2), either on the last day of the FMD week or one week after refeeding. During the FMD cycle, both males (Fig. 2A) and females (Fig. 2B) exhibited a significant decrease in blood glucose levels, which was restored during refeeding.

In physiological conditions, the concentration of β-HB is very low (≤5.4 mg/dL), whereas during nutritional ketosis, this concentration increases to over 9 mg/dL [37, 38]. β-HB levels were significantly different during the FMD cycles in both sexes (males, *p* = 0.0012; females, *p* = 0.0002), although there was high variability among the samples. Circulating levels of β-HB increased during the FMD week, reaching “mild” ketosis in males (9.54±5.58 mg/dL; Fig. 2C) and females (11.52±6.48 mg/dL; Fig. 2D). After refeeding, blood glucose and β-HB levels in the VCM+FMD group were comparable to those of the VCM+SD group for both males (glucose: 133.56 ± 17.55 mg/dL, ketones: 2.16 ± 2.52 mg/dL) and females (glucose: 132.89 ± 20.32 mg/dL, ketones: 0.36 ± 0.72 mg/dL). Overall, these results suggest that FMD affected the metabolic state and induced a fasting-like environment in VCM mice.

### Effect of FMD on body weight and plasma FFA levels in VCM mice

**Fig. 3:**
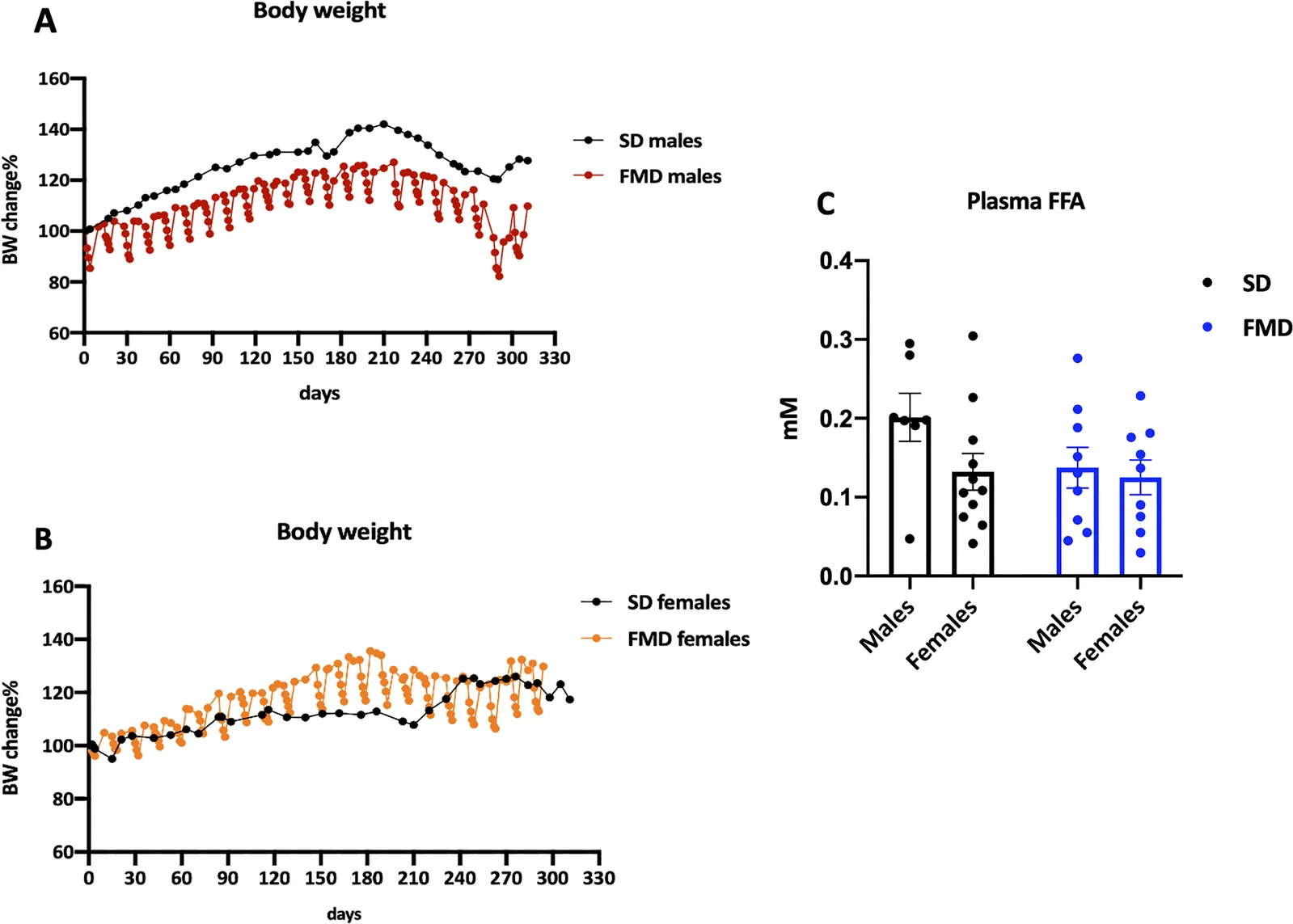
Body weight and plasma free fatty acid (FFA) levels in VCM. **A-B**) body weight change in VCM+SD and VCM+FMD males (**A**) and females (**B**). Average body weight is expressed as a percentage of the initial body weight of the experimental animals. Only mice showing macroscopic tumour masses at necropsy or that died prematurely are represented. VCM mice fed with SD males (*n* = 8) and females (*n* = 6). VCM mice fed with FMD males (*n* = 12) and females (*n* = 7). **C**) histograms reporting plasma FFA levels in VCM+SD and VCM+FMD mice, stratified for sex. Plasma FFA levels are expressed in mM and measured at the endpoint during refeeding. VCM+SD males (*n* = 7) and females (*n* = 11); VCM+FMD males (*n* = 9) and females (*n* = 9). Significance of the differences in plasma FFA levels by two-way Anova test. When comparing males and females within the SD group a student’s *t*-test was applied

Fasting and CR have been popular strategies for treating obesity and protecting against metabolic syndrome [39, 40]. Indeed, alternate-day fasting in rodent models of obesity has been shown to decrease total plasma cholesterol and triglyceride concentration [40]. The monitoring of VCM animal body weight showed significant differences between male and female animals. Male FMD-fed mice were characterized by a lower weight than male VCM+SD animals (Fig. 3A), with a statistical reduction after 6 months of intervention and lasting both during the FMD administration and during the SD refeeding (Figure S1). Conversely, the weight of female mice slightly increased in the FMD group (Fig. 3B), with only a couple of statistically significant points, again after 6 months of nutritional intervention (Figure S1). Notably, during the FMD week, both males and females exhibited reduced energy intake, which returned to the same levels as those of the controls after several days of refeeding (Figure S2).

To investigate the effect of FMD on circulating FFA, VCM mice plasma was analysed at the endpoint of the refeeding week. FMD-fed animals showed similar plasma FFA to the SD-fed in both males and females (Fig. 3C). However, when comparing only males and females in the SD group, males showed a significant increase (*p* = 0.046) in FFA compared to females.

### Modulation of gut microbiota by FMD

Since the possible beneficial effects of dietary restrictions on the gut microbiota, including FMD, have been previously reported [25, 26], faecal samples were collected at two time points to evaluate the gut microbiota composition after FMD dietary regime (Fig. 1). Specifically, shotgun metagenomic sequencing was performed on faecal samples collected for both groups at the age of six months (T1), before the frankly appearance of tumours, and at twelve months (T2), the time in which in the majority of the mice tumours are likely to be present (for the VCM+FMD fed group one week after refeeding).

**Fig. 4:**
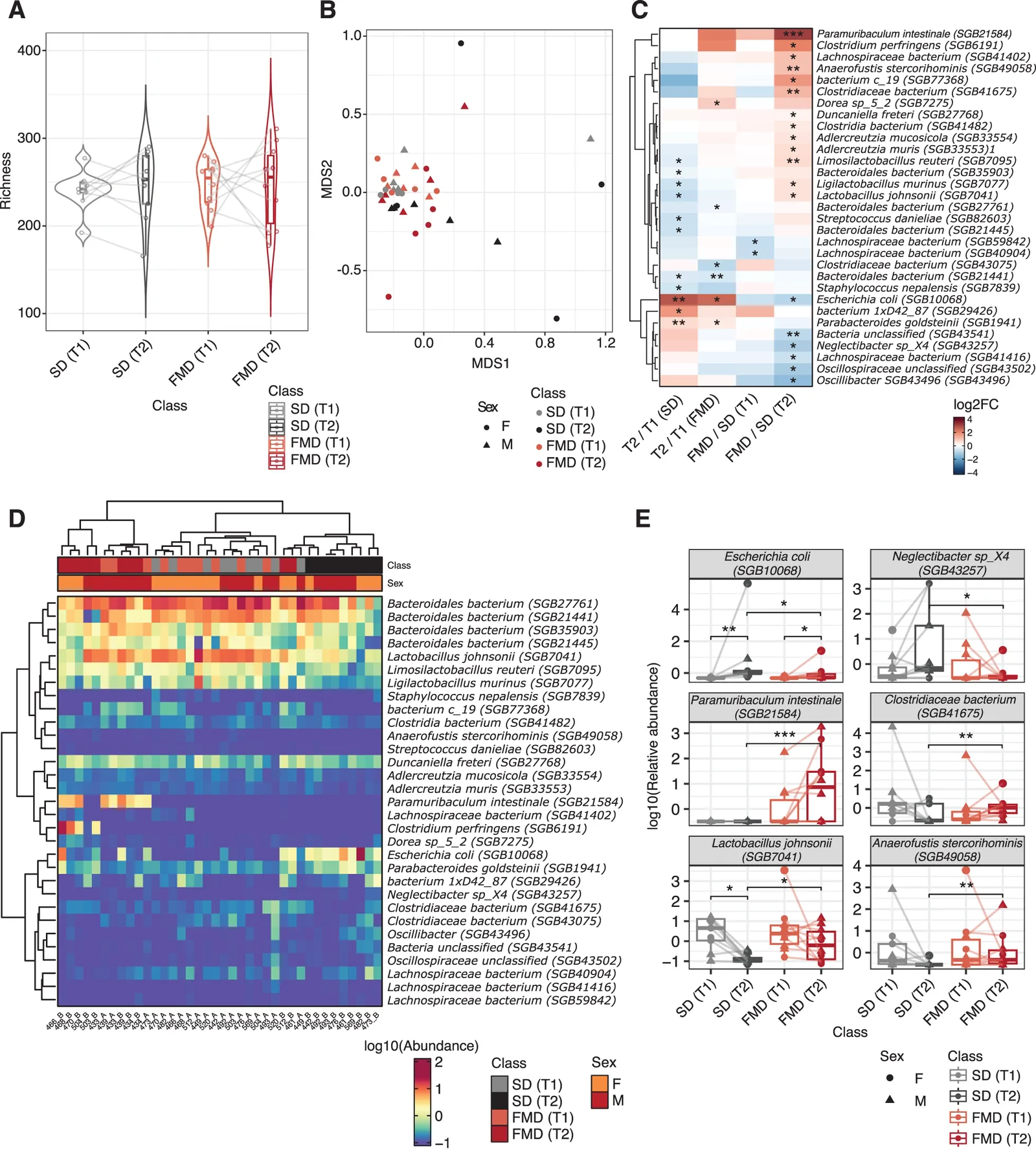
Analysis of the faecal microbial composition among VCM+SD and VCM+FMD mice stool samples collected at T1 and T2. **A**) violin plots of the microbial richness computed for each study group at the two time points. *p*-value by Wilcoxon rank-sum test. **B**) multidimensional scaling (MDS) plots representing the beta diversity among samples from SD and FMD mice at T1 and T2. VCM+SD males and females (*n* = 9) and VCM+FMD males and females (*n* = 10). **C**) heat map reporting the species-level genome bins (SGBs) with significantly different abundances among the investigated study groups. The colour code represents the log2FC of SGB abundances as computed in the comparisons reported in columns. Significance of the differences by SIAMCAT test: *p < 0.05; **p < 0.01; ***p < 0.001. Paired analysis was performed for the T2 vs T1 comparisons. **D**) heat map with relative abundance levels (reported in log10) of the differentially abundant SGBs. Heat map columns are annotated with the study groups and sex information. VCM+SD (*n* = 9) and VCM+FMD (*n* = 10). **E**) box plots reporting the log10 relative abundances of six representative bacterial species in the VCM+FMD and VCM+SD samples. Significance of the differences by Wilcoxon rank-sum test: *p < 0.05; **p < 0.01

No significant changes in microbial richness (number of detected species) were observed between either T1 and T2 or VCM+SD and VCM+FMD samples (Fig. 4A). Similarly, no significant differences were observed among groups for the other two alpha diversity metrics, the Shannon Index and Inverse Simpson index (Figure S3A-C and Supplementary Table 2A). On the other hand, the VCM+SD group at T2 showed a higher beta diversity (measuring the differences in microbial composition between samples) than T1 samples from the same animals but also than VCM+FMD in all the time points, with the time variable significantly associated with the beta diversity (PERMANOVA *p* = 0.001; Fig. 4B and Supplementary Table 2B).

Profiling of microbial abundance in the analysed stools yielded an average of 245 ± 34 SGBs per sample. Differential analysis of the relative abundances by microbial phyla revealed an increase in Firmicutes and Proteobacteria at T2 in both groups (Figures S2D and E). Differentially abundant SGBs, time or diet-dependent, are reported in Figure 4C and Supplementary Table 2C. Observing the VCM+SD group over time, some species, such as *E. coli (SGB10068)* and *Parabacteroides goldsteinii (SGB1941),* were more abundant (paired Wilcoxon test *p* < 0.05) at T2, while other microbes, such as *Bacteroidales bacterium (SGB21441)* and *Lactobacillus johnsonii (SGB7041)* decreased at T2. Similarly, in the VCM+FMD group, the abundance of the same species increased when animals approached the endpoint. Among the differentially abundant SGBs, 13 were unclassified at species levels, including four *Bacteroidales bacterium* SGBs. Notably, these SGBs were characterized by the highest levels among the differential SGBs, particularly *Bacteroidales bacterium (SGB27761 and SGB21441)*. Based on the Genome Taxonomy Database (GTDB) annotations, these *B. bacterium* SGBs were also defined as *CAG-485 sp002493045* (*SGB27761*), *UBA7173 sp001689685* (SGB21441), *Muribaculum sp002473395* (*SGB35903*), and Duncaniella sp001689575 (SGB21445).

The clustering analysis revealed a clear separation between the samples at T2, highlighting some SGBs with a specifically higher abundance, such as *E. coli (SGB10068)* in VCM+SD animals and *Paramuribaculum intestinale (SGB21584)* in VCM+FMD (Fig. 4D). Moreover, significant differences in the abundance of 19 species were observed between SD and FMD at T2. Details of some of these taxa are reported in Fig. 4E. For instance, the abundance of *E. coli (SGB10068)* increased at T2 in both VCM+SD and VCM+FMD; however, this increase was significantly smaller in animals subjected to FMD cycles. Similarly, a rise in *Neglectibacter sp. X4 (SGB43257)* was not observed in mice subjected to the FMD regimen at T2 but was found in animals receiving the SD. Conversely, *P. intestinale (SGB21584)* and *Clostridiaceae bacterium (SGB41675)* abundances increased in VCM+FMD at T2 compared with the VCM+SD at the same timepoint. Furthermore, the FMD prevented the general decrease in the abundance of *L. johnsonii (SGB7041)* and *Anaerofustis stercorihominis (SGB49058)* at T2.

Given the known relevance of *E. coli* colonization in CRC development, and to gain a better understanding of the possible influence of animal sex, we evaluated the relative abundance of these bacteria in the two sex cohorts. An increased abundance of *E. coli (SGB10068)* was observed in VCM+SD animals at T2 compared to VCM+FMD mice (Fig. 4E). Since among the *E. coli* strains those encoding colibactin (*pks*+) are involved in CRC development [41], we explored the levels of 55 genes in the *pks* island annotated in UniProt (Supplementary Table 2D) using HUMAnN, and we found a total absence of these genes in all analysed samples.

### FMD induced alterations on microbial metabolic pathways

**Fig. 5:**
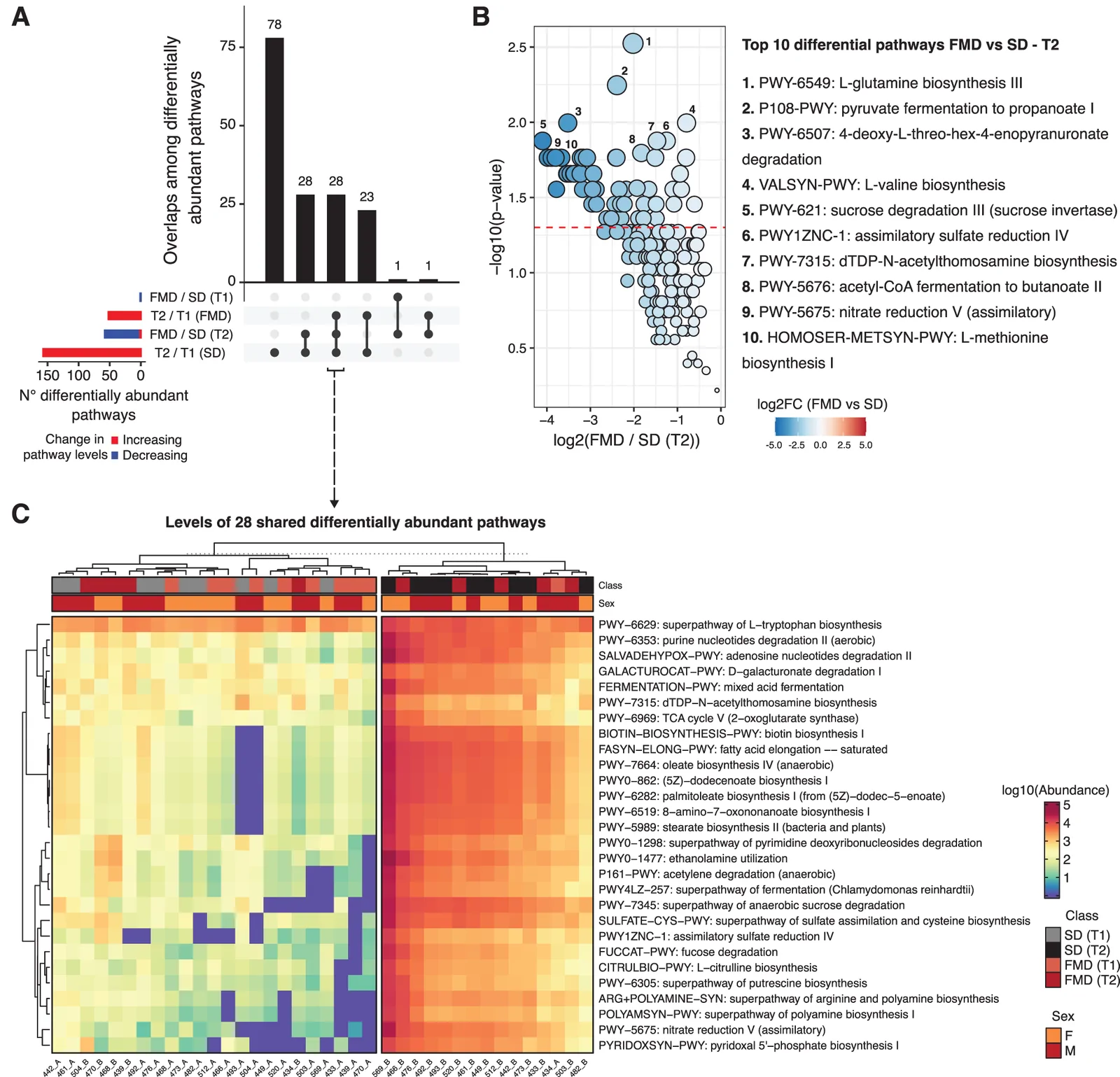
**A**) Upset plot of the number of microbial pathways associated with significantly different levels between study time points and VCM+SD and VCM+FMD animals. The color-code of the horizontal bars represent the espression trend of the differentially abundant pathways obtained in each comparison. The vertical arrow indicates the set of shared differentially abundant microbial pathways whose levels is reported in the panel C. **B**) volcano plot reporting the changes in microbial pathway levels between T2 time points of VCM+SD and VCM+FMD animals, considering 157 pathways observed as significantly modulated between T2 and T1 time points of VCM+SD mice. *p*-value by Wilcoxon rank-sum test. The red dashed lines represent the *p*-value threshold of 0.05. The identifier of the top 10 significantly differentially abundant pathways is reported. **C**) heatmap and clustering analysis of the levels of 28 microbial pathways identified as commonly differentially abundant between T1 and T2 samples of both dietary groups and in the comparison of T2 samples from SD and FMD animals

The analysis of microbial metabolic pathways enriched in this study showed significant differences among the investigated groups (Supplementary Table 2E). Specifically, among the 234 microbial pathways analysed, 157 and 52 were associated with a substantial (paired Wilcoxon test, *p* < 0.05) modulation in VCM+SD and VCM+FMD, respectively, with all increasing their levels at T2 (Figure 5A). Notably, the levels of all 157 pathways modulated in VCM+SD, including the 52 pathways observed in VCM+FMD, decreased at T2 in VCM+FMD animals (Figure 5B). The most significantly decreased pathways in VCM+FMD were *L-glutamine biosynthesis III (PWY-6549)*, *pyruvate fermentation to propanoate I (P108-PWY)*, *4-deoxy-L-threo-hex-4-enopyranuronate degradation (PWY-6507)*, *L-valine biosynthesis (VALSYN-PWY)*, *5-aminoimidazole ribonucleotide biosynthesis II (PWY-6122), superpathway of 5-aminoimidazole ribonucleotide biosynthesis (PWY-6277), pyrimidine deoxyribonucleotides de novo biosynthesis IV (PWY-7198)*.

Among them, 28 were associated with levels that were significantly higher at T2 of both animal groups, but significantly lower at T2 in VCM+FMD mice compared to those in VCM+SD animals (Figure 5C).

A species-level analysis of bacteria contributing to these pathways showed that, for all of them, the difference was mainly driven by the lower abundance of *E. coli* in FMD samples, and it was significantly different (*p* < 0.05, comparing FMD and SD T2 samples) for the *superpathway of sulfate assimilation and cysteine biosynthesis (SULFATE-CYS-PWY), fatty acid elongation – saturated (FASYN-ELONG-PWY), ethanolamine utilization (PWY0-1477);* details can be found in Supplementary Table 2F. Another species contributing to these pathways was *Enterococcus faecalis* (7 out of 28 pathways), whose levels increased only in SD animals at T2. At T2 in FMD animals, this bacterium was associated with a significantly lower contribution to the *superpathway of pyrimidine deoxyribonucleosides degradation (PWY0-1298)* and *palmitoleate biosynthesis I (from (5Z)-dodec-5-enoate; PWY-6282).*

A comparable result was observed by exploring the Gene Ontology scores computed based on the microbial gene abundances. Specifically, the score of 2,348 terms was significantly different in at least one comparison, of which 1,626 were significantly different in the comparison between T2 samples from SD and FMD animals (Supplementary Table 2 G). Notably, among them, 1,606 (98.8%) were associated with a lower score in FMD animal samples, including *GO:0008519 (ammonium transmembrane transporter activity), GO:0006567 (threonine catabolic process)*, *GO:0004455 (ketol-acid reductoisomerase activity*) as the most significantly decreasing terms (*p* < 0.01). Conversely, the 20 terms with higher scores in T2 FMD samples include *GO:0047150 (betaine-homocysteine S-methyltransferase activity)*, *GO:0004623 (phospholipase A2 activity)*, and *GO:0050482 (arachidonic acid secretion)* as the most significant terms (*p* < 0.01).

## Discussion

The FMD is an emerging nutritional regimen that focuses on implementing specific nutrient combinations and complex food compositions to improve healthspan and lifespan, as well as optimize therapies [42]. Most studies investigating FMD in animal models of cancer have focused on its potential to improve the efficacy of antineoplastic treatments by modulating cancer and host metabolism or enhancing antitumour responses [43–46]. The current study, adopting a mouse model of spontaneous cancer with late and low pace tumour onset, aimed at understanding the impact of long-term FMD on host health and microbiota.

FMD did not prevent tumour development suggesting that, at least in this model with constitutive *Msh2* deletion, FMD alone is insufficient to block tumorigenesis. In accordance with the literature [47], the administration of FMD in VCM animals led to metabolic changes characterized by a marked decrease in blood glucose and an increase in β-HB levels. Notably, our study has provided interesting data regarding the sex-based differences in body weight change upon FMD, with males benefiting significantly more from FMD in limiting body weight gain. Several biological factors may explain the dimorphic effect, likely impinging on whole-body metabolism, such as hormone levels and differences in adipose tissue distribution and function [48]. Females exhibit higher thermogenic activity than males, while males generally have higher resting energy expenditure (REE) [49]. This difference in REE may explain why males tend to lose more body weight during fasting-mediated diet-dependent calorie restriction (FMD-dependent CR). Finally, CR may help prevent cardiometabolic risk factors [50, 51], indeed, elevated plasma FFA levels were observed exclusively in SD-fed male mice, likely due to elevated adiposity.

The gut microbiota undergoes significant changes in the course of cancer and intestinal inflammatory diseases [26, 52]. More specifically, increased Proteobacteria and the Firmicutes/Bacteroides ratio, along with a decrease in *Lactobacilli* [53] and *Muribaculaceae* [54], have been observed in humans and mice with CRC. In this study, we observed that variations in beta diversity may indicate a potential connection between tumour progression and microbial diversity in SD-fed mice. FMD has previously demonstrated the ability to reduce intestinal inflammation and promote the colonization of beneficial gut microbiota in an experimental model of inflammatory bowel disease [25]. In the present study, we showed that a long-term intervention with FMD may prevent the microbiota shift observed during CRC progression, increasing the abundance of species belonging to the probiotic bacterial families *P. intestinale* (a species of *Muribaculaceae)*, *P. goldsteinii, and L. johnsonii*, crucial for intestinal health [55, 56]. *Lactobacillus* and *Bifidobacterium* strains have been implicated, among others, in the suppression of NF-κB activation and IL-6 secretion, as well as the production of short-chain fatty acids (SCFAs), which exert a protective action on the intestinal barrier against inflammatory pathologies [57]. In addition, as demonstrated by Luo et al. (2024) [26], FMD promotes the expansion of *L. johnsonii*, which plays a role in modulating the immune microenvironment in mice subcutaneously implanted with MC38 cells, enhancing CD45+ and CD8+ T cell populations. Bacterial species such as *E. coli* have been identified as CRC-promoting bacteria [12, 16, 17]. Chronic exposure of healthy human intestinal organoids to genotoxic *pks*^+^ *E. coli* promoted mutagenesis by inducing specific single-base substitutions (SBS-*pks*) and small insertions and deletions (ID-*pks*) mutational signatures [58]. Although *pks+ E. coli* has been found in both normal and cancerous intestinal tissues, CRC patients have a higher abundance compared to healthy individuals [16, 17]. In the present study, the abundance of *E. coli* increased in the late sampling (T2), especially in the males of the SD group; however, no genes related to the *pks* island were detected. Laboratory mice are typically bred and raised in isolated, germ-free environments that do not naturally harbour specific bacterial strains, such as *pks*+ *E. coli*. To study the role of these bacteria in cancer [59] and other diseases [60], animals are often deliberately colonized with *pks*+ *E. coli*. In the current study, it remains unclear whether the increase in *E. coli* is a result of changes caused by the tumour microenvironment or if it is an earlier event, potentially acting as a promoter of CRC development. Further research is needed to determine the underlying cause.

The differences in microbial species observed at T2 resulted in the upregulation of microbial metabolic pathways in the SD group, likely resulting in dysbiotic microbiota-derived metabolites. This suggests that unrestricted feeding in the SD group may have promoted more active microbial metabolism over time compared to FMD, potentially having a negative impact on intestinal health. In contrast, FMD may promote a selection for a more specialized microbial population that is resilient to reduced gut nutrient availability, which aligns with the increased beta diversity observed in the SD group as it approaches the endpoint.

Among the most significantly modulated metabolic pathways between the two regimens are those involved in amino acid and nucleotide synthesis, which potentially contribute to the production of alternative energy sources and biosynthetic molecules essential for tumour growth in the SD group. Conversely, the increased metabolic pathway for producing SCFAs (*pyruvate fermentation to propanoate I*) observed in the SD group may counteract CRC proliferation [61], as observed for propionate production in the gut, which has been shown to induce apoptosis in CRC cells in 3D spheroid culture models [62]. Despite these findings, the activity of these metabolic pathways cannot be determined, requiring further validation through metabolomics and in vivo testing of the antiproliferative action.

Overall, our findings indicate that FMD differentially impacts the body weight of animals in a sex-dimorphic manner, highlighting the importance of considering sex even in the context of nutritional regimen response, as it is already emerging for humans [63]. Consequently, it is crucial to carefully consider and address sex-based differences in dietary studies, as these factors may confound the results and limit the generalizability of the findings. Furthermore, this study suggests that chronic intervention with FMD may slow down the metabolic activity of the intestinal microbial population and prevent the microbiota modulations observed during the development of CRC. However, establishing a direct cause-and-effect relationship between this modulation and specific aspects of CRC development or progression remains challenging, especially given the lack of a macroscopic impact on the tumour.

While this study provides novel insights, several limitations should be acknowledged. Stool samples have been used as non-invasive proxy for studying the gut microbiota associated with tumour development. However, such samples reflect mainly the composition from the large intestine and only partly from the small intestine, where most of the tumours were found. However, such experimental design was chosen given the impossibility to collect paired longitudinal samples from the small intestine an even in the attempt to obtain the higher translational value for future clinical practice. Baseline (T0) microbiome data were not collected prior to dietary intervention. However, as shown in our data, minor differences in microbial abundance were observed between groups at T1, before the onset of overt pathological manifestations, suggesting that this time point may approximate a baseline condition. In this context, the microbiome changes observed in the later stages are more likely to be related to disease progression and its interaction with dietary intervention. Although maternal effects may influence the composition of the gut microbiota, animals were randomly assigned to experimental groups and originated from multiple litters, thereby minimizing potential bias related to maternal origin. Consistently, the analysis of maternal dependency on beta diversity among samples at either T1 or T2 (Figure S4) did not provide support to such hypothesis. During the dietary intervention, the animals were housed in groups to reduce stress, however, this prevented accurate monitoring of individual food intake and may have contributed to interindividual variability in metabolic parameters. Nevertheless, feeding procedures and sampling times were rigorously standardized to limit experimental variability, and food was provided in a manner that reduced competition among the animals. Importantly, our data reveal a significant metabolic switch between groups, supporting the effectiveness of the experimental design. Finally, this study was not specifically designed to assess the impact of FMD on tumour incidence or progression. Addressing this question would require a dedicated experimental design with sufficient statistical power and a more detailed oncological evaluation.

Despite these limitations, this study represents the first longitudinal application of FMD in a spontaneous and heterogeneous model of hereditary intestinal cancer, providing a translational framework that may serve as a basis for future mechanistic and therapeutic studies.

In conclusion, while there is evidence suggesting that FMD influences the gut microbiota, the potential implications of these alterations for cancer patients remain to be fully understood. Furthermore, the use of FMD is not recommended for individuals with high susceptibility to malnutrition and cachexia, emphasizing the need for specialized monitoring and prudent implementation. Ultimately, although FMD cannot replace standard cancer treatment, it may serve as a complementary strategy to promote beneficial bacterial strains, thereby contributing to a more balanced gut microbiota, and potentially acting as an alternative to probiotics, as part of a multimodal approach targeting the overall health of populations at risk of developing or affected by intestinal cancers.

## Electronic supplementary material

Below is the link to the electronic supplementary material.

Supplementary Material 1

Supplementary Material 2

Supplementary Material 3

Supplementary Material 4

Supplementary Material 5

Supplementary Material 6

## Data Availability

All the raw data are available from the corresponding author upon request. Metagenomic sequencing data have been deposited at NCBI, with official ID pending.

## Declarations

### Generative AI in scientific writing

No AI tool has been used for the writing of this manuscript.
