# Supplementary material for: Fasting-mimicking diet counteracts gut microbial dysbiosis in experimental lynch syndrome
Source: Cancer Metab. 2026 Jun 30;14:31. doi: 10.1186/s40170-026-00446-1 (PMC13591788; doi:10.1186/s40170-026-00446-1)
Supplement: Supplementary file 1 [file 40170_2026_446_MOESM1_ESM.pdf]

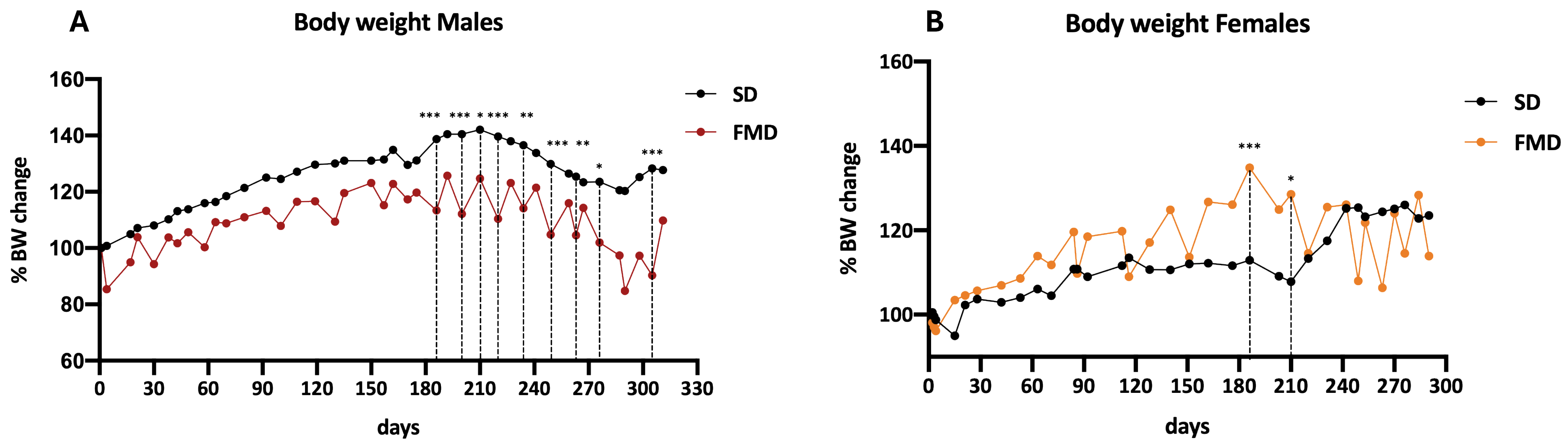

Body weight change in VCM+SD and VCM+FMD males (**A**) and females (**B)**. Average body weight is expressed as a percentage of the initial body weight of the experimental animals. Only mice showing macroscopic tumour masses at necropsy or that died prematurely are represented. VCM mice fed with SD males (n=8) and females (n=6). VCM mice fed with FMD males (n=12) and females (n=7). Significance of the differences by Two-way Anova test.
