# Supplementary material for: Fasting-mimicking diet counteracts gut microbial dysbiosis in experimental lynch syndrome
Source: Cancer Metab. 2026 Jun 30;14:31. doi: 10.1186/s40170-026-00446-1 (PMC13591788; doi:10.1186/s40170-026-00446-1)
Supplement: Supplementary file 2 [file 40170_2026_446_MOESM3_ESM.pdf]

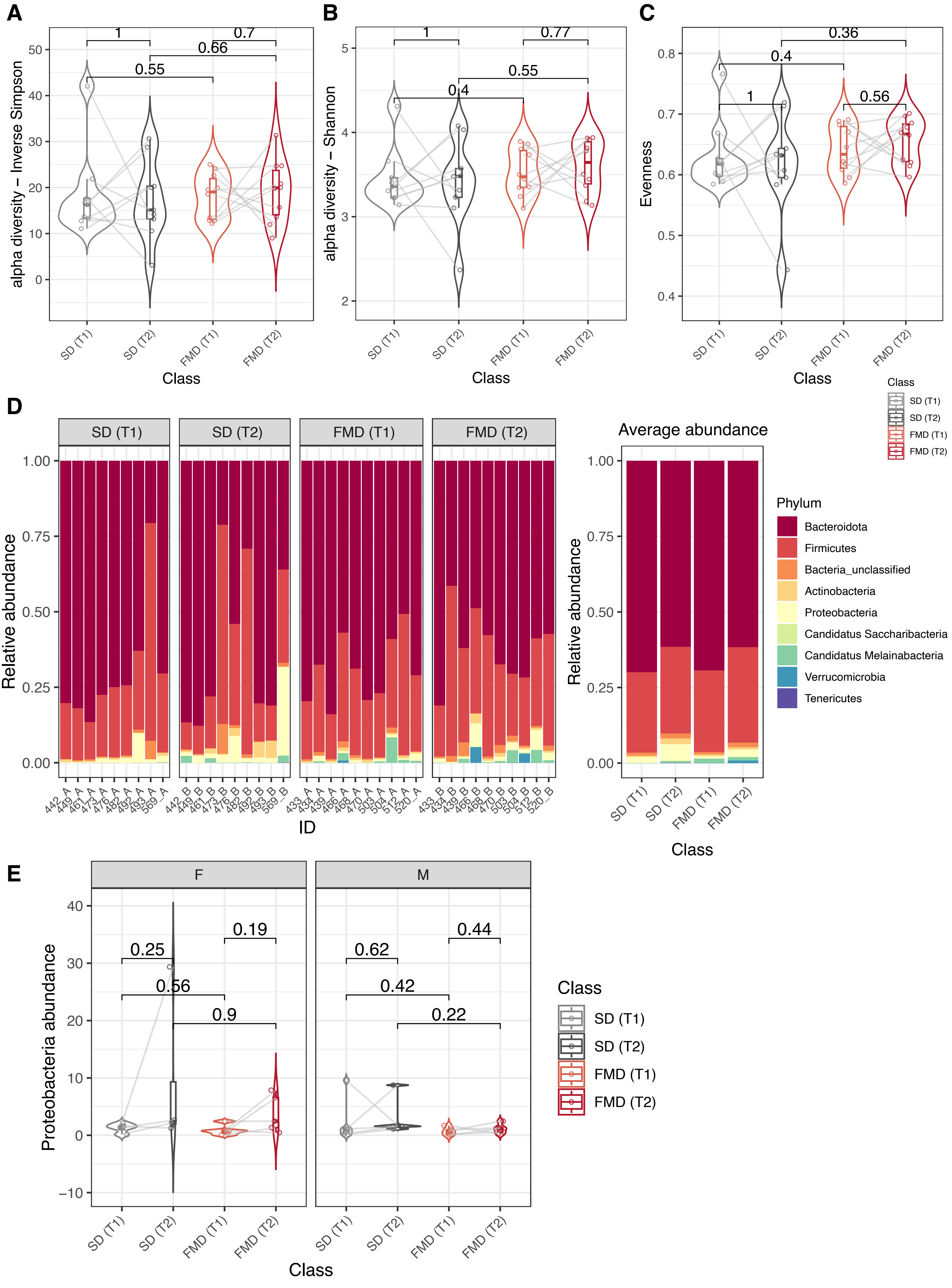
A
alpha diversity – Inverse Simpson
50
40
30
20
10
0
1
0.7
0.66
0.55
SD (T1)
SD (T2)
FMD (T1)
FMD (T2)
Class
B
alpha diversity – Shannon
5
4
3
2
1
0.77
0.55
0.4
C
Evenness
0.8
0.7
0.6
0.5
0.4
0.36
0.4
1
0.56
Class
SD (T1)
SD (T2)
FMD (T1)
FMD (T2)
D
SD (T1)
SD (T2)
FMD (T1)
FMD (T2)
Relative abundance
1.00
0.75
0.50
0.25
0.00
442_A
449_A
461_A
473_A
476_A
482_A
492_A
493_A
569_A
442_B
449_B
461_B
473_B
476_B
482_B
492_B
493_B
569_B
433_A
434_A
439_A
466_A
468_A
470_A
503_A
504_A
512_A
520_A
433_B
434_B
439_B
466_B
468_B
470_B
503_B
504_B
512_B
520_B
ID
Average abundance
Phylum
Bacteroidota
Firmicutes
Bacteria_unclassified
Actinobacteria
Proteobacteria
Candidatus Saccharibacteria
Candidatus Melainabacteria
Verrucomicrobia
Tenericutes
E
F
M
Proteobacteria abundance
40
30
20
10
0
−10
0.25
0.19
0.56
0.9
0.62
0.44
0.42
0.22
Class
SD (T1)
SD (T2)
FMD (T1)
FMD (T2)
