# Supplementary material for: Fasting-mimicking diet counteracts gut microbial dysbiosis in experimental lynch syndrome
Source: Cancer Metab. 2026 Jun 30;14:31. doi: 10.1186/s40170-026-00446-1 (PMC13591788; doi:10.1186/s40170-026-00446-1)
Supplement: Supplementary file 3 [file 40170_2026_446_MOESM4_ESM.pdf]

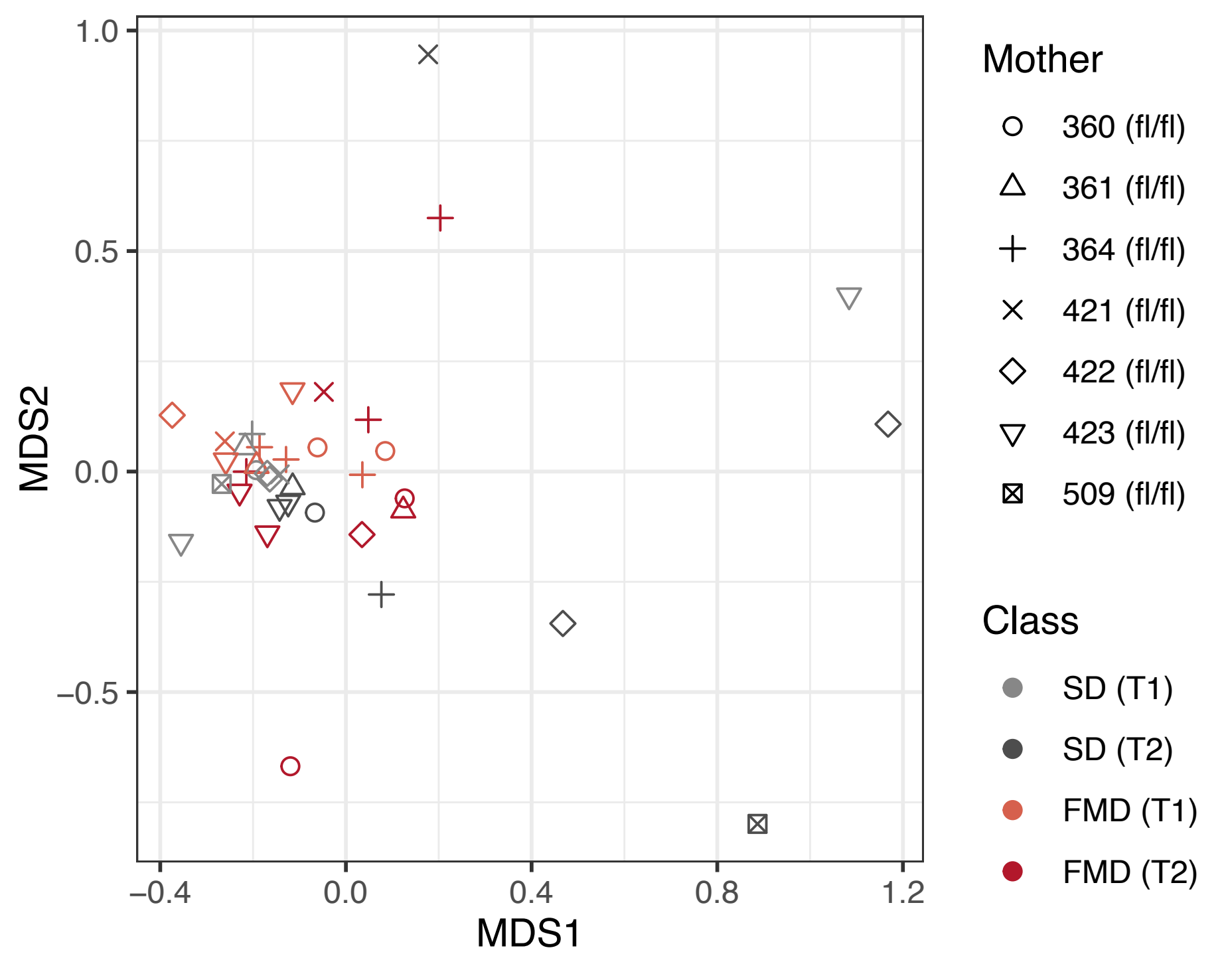

MDS2
MDS1
1.0
0.5
0.0
−0.5
−0.4
0.0
0.4
0.8
1.2
Mother
360 (fl/fl)
361 (fl/fl)
364 (fl/fl)
421 (fl/fl)
422 (fl/fl)
423 (fl/fl)
509 (fl/fl)
Class
SD (T1)
SD (T2)
FMD (T1)
FMD (T2)
